# Exergy Analyses of Onion Drying by Convection: Influence of Dryer Parameters on Performance

**DOI:** 10.3390/e20050310

**Published:** 2018-04-25

**Authors:** María Castro, Celia Román, Marcelo Echegaray, Germán Mazza, Rosa Rodriguez

**Affiliations:** 1 Instituto de Ingeniería Química—Facultad de Ingeniería, National University of San Juan (UNSJ), Libertador 1109 (O), 5400 San Juan, Argentina; 2 Instituto de Investigación y Desarrollo en Ingeniería de Procesos, Biotecnología y Energías Alternativas PROBIEN, National University of Comahue (CONICET-UNCo), Buenos Aires 1400, 8300 Neuquén, Argentina

**Keywords:** onion drying, exergy analysis, exergetic improvement potential rate, sustainability index

## Abstract

This research work is concerned in the exergy analysis of the continuous-convection drying of onion. The influence of temperature and air velocity was studied in terms of exergy parameters. The energy and exergy balances were carried out taking into account the onion drying chamber. Its behavior was analyzed based on exergy efficiency, exergy loss rate, exergetic improvement potential rate, and sustainability index. The exergy loss rates increase with the temperature and air velocity augmentation. Exergy loss rate is influenced by the drying air temperatures and velocities because the overall heat transfer coefficient varies with these operation conditions. On the other hand, the exergy efficiency increases with the air velocity augmentation. This behavior is due to the energy utilization was improved because the most amount of supplied energy was utilized for the moisture evaporation. However, the exergy efficiency decreases with the temperature augmentation due to the free moisture being lower, then, the moisture begins diffusing from the internal structure to the surface. The exergetic improvement potential rate values show that the exergy efficiency of onion drying process can be ameliorated. The sustainability index of the drying chamber varied from 1.9 to 5.1. To reduce the process environmental impact, the parameters must be modified in order to ameliorate the exergy efficiency of the process.

## 1. Introduction

Thermodynamics plays an important role in analyzing the energy efficiency of industrial processes. The used energy through a process is important, and its utilization optimization is substantial considering the environmental and economic aspects. Through exergy analysis, it is possible to identify potential savings taking into account the operating conditions. Exergy is defined as the maximum work quantity produced from a matter flow, heat, or work during the equilibrium is reached with the environment taken as reference. Exergy analysis is also a useful methodology to establish strategies in the design and operation of industrial processes when the energy use must be optimized. In recent years, exergy analysis has been amply used to evaluate thermal system yields.

Energy balance is the traditional approach to evaluate various energy conversion processes. The energy balance, the first law of thermodynamics, can calculate heat losses, but it does not provide information about the optimal energy transformation.

This objective can be reached in the exergy analysis, due to the second law of thermodynamics establishing that all the input energy in the system cannot be transformed into useful work. In this analysis, the first and second laws of thermodynamics are used. Exergy is not topic for conservation law, but the exergy is consumed or destroyed due to irreversibilities in any process [1].

On the other hand, considering the drying process, the objective is to use the minimum amount of energy to obtain the maximum moisture removal and to achieve the desired product conditions [2]. This process is widely used in various industries—such as food, chemical, wood, biotechnology, polymer, ceramics, pharmaceutical, and bio-energy industries—consuming the large amount of energy. It is generally used to remove moisture or liquid from a wet solid, converting them to the vapor state. In most drying operations, the water is evaporated liquid and air is normally used as a purge gas. Due to the high prices and shortage of energy, environmental concerns and decreasing fossil-fuel recourses, the optimum application of energy and the methods of energy consumption management are very important for enterprise sustainability.

Bardy et al. [3,4] applied the thermodynamic analysis proposed by [5] to the drying of methylcellulose gel and mango fruit in forced convection drying with and without electrohydrodynamic enhancement.

Boulemtafes-Boukadoum and Benzaoui [6] carried out an energy and exergy analysis of a solar drying process for mint. They used an indirect type, passive dryer without extra energy and discontinuous operation. These researchers quantified the solar energy received by a solar heater available for drying, using the energy analysis. The energy losses during the drying process were estimated through the exergy. Rabha et al. [7] realized energy and exergy analyses of the solar drying processes of ghost chili pepper and ginger, using a forced convection solar tunnel dryer integrated with a shell and tube based latent heat storage module. They concluded that the thermal efficiencies of the first and the second solar air heaters varied between 22.10% and 40.24% and 9.64% and 19.50%, respectively. When the ghost chili was dried, the average exergy efficiency of the drying chamber was equal to 63%, and it was equal to 47%, while the ginger was dried. The high exergy efficiency was registered in the last few hours of the drying process of the consecutive drying days.

Brasil Maia et al. [8] carried out a thermodynamic analysis of the drying process of bananas in a small-scale solar updraft tower. They proposed a model based on the first and second laws of thermodynamics, using the ambient conditions and airflow parameters data obtained in the experimental prototype. They concluded that the incident solar radiation plays an important role on the drying process of bananas, the higher the solar radiation, the higher the exergy rates.

Azadbakht et al. [9] analyzed the energy and exergy loss in the drying of eggplant using a fluidized bed dryer. They investigated the effects of temperature, flow rate of drying air, and sample size on energy consumption and exergy losses. Their results showed that the minimum energy consumption and exergy losses occurred at a diameter of 13 mm, air flow rate of 3 m s^−1^, and temperature of 313 K. The results demonstrated that higher temperature, air flow rate, and eggplant samples’ lesser diameter increased energy consumption.

The objective of this study is to present energy and exergy analyses of onion drying at different conditions of air temperature and velocity considering a continuous-convection process. The proposed model does not include properties related to the material particles to be dried, in this case, onions. The studied system is the drying chamber, considered a black box.

## 2. Methodology

In this work, the drying air parameters’ influence, as temperature and velocity, in an onion drying system, was studied, using the exergy analysis. A thermodynamic model was proposed based on the mass, energy and exergy balances, considering the onion drying chamber. The exergy performance based on exergy efficiency, exergy loss, exergetic improvement potential (*IP*), and sustainability index (*SI*), were analyzed. The initial humidity of the air was set as 0.01 kg water/kg dry air. The temperature and air velocity were considered as independent variables. The drying process was evaluated for 50, 60, 70, and 80 °C and 0.5, 1, and 2 ms^−1^, respectively. Table 1 shows the variation of air flow rate at different conditions.

To know the behavior of onion during the dying, fresh slices of onion of the Angaco Inta variety were used to carry out the drying experiment. A laboratory-scale dryer with drying air temperature and velocity control was used. The experiments were carried out until final water mass fractions equal to 0.05. The initial water mass fractions were determined experimentally, and it is equal to 0.89. Each set of the experiments replicated three times, and the average values were used. The drying rates of onions were equal to 0.010, 0.012, 0.014, and 0.018 g water/g dry matter min for 50, 60, 70, and 80 °C respectively. The experiments were carried out in batch system and the obtained results were extended to a continuous dryer.

In order to write balanced equations for the different components of the drying chamber, the following assumptions have been made:

The drying chamber production was considered equal to 1 kg of onion/h.

The initial temperature of onion was considered equal to 25 °C.

The heat capacity of wall material of drying chamber is neglected.

There is no stratification in the air temperature of the drying chamber. 

The absorptivity and heat capacity of enclosed air is neglected.

### 2.1. Energy Balance

Considering the input and output energy flows through the system, the energy balance Equation can be written as [10]

(1) $$Q=m_{dp}\cdot {\hat{H}}_{dp}+m_{ma}\cdot {\hat{H}}_{ma}+m_{wo}\cdot {\hat{H}}_{wo}-m_{da}\cdot {\hat{H}}_{da}-m_{wp}\cdot {\hat{H}}_{wp}-m_{wi}\cdot {\hat{H}}_{wi}$$

where *Q* is the heat transferred to or from the walls of the drying chamber, in this case adiabatic conditions were considered so it takes the value zero. $\hat{H}$ is the enthalpy content per mass unit of each stream, expressed in kJ/kg, and *m* is the mass flow rate of each stream, expressed in kg/h. The subscripts *dp*, *ma*, *da*, *wi*, and *wo* correspond to the properties of dry product, humid air, dry air, contained water in the input product, and water contained to the output product, respectively.

### 2.2. Entropy Balance

The general entropy balance was expressed as function of the mass flows (the input and output flows), their entropies, and the transferred heat through the walls of the drying chamber. This heat can have negative or positive signs, depending upon the heat transferred to or from the control volume, in this case the dryer.

### 2.3. Exergy Balance

The exergy losses in the drying chamber are associated with the exergy losses with the air left by the dryer and the exergy losses from the walls with the heat loss and the exergy losses in the product. The exergy balance was developed based on the general form of the exergy flow Equation for steady-state systems. The effect of kinetic and potential energies was considered negligible. In the drying chamber, the work is equal to cero. Therefore, the transfer of exergy due to the transfer of work is zero, too. Analogous to the energy balance, the balance of exergy to the system is [11]

(2) $$m_{dp}\cdot \varepsilon_{dp}+m_{ma}\cdot \varepsilon_{ma}+m_{wo}\cdot \varepsilon_{wo}=m_{da}\cdot \varepsilon_{da}+m_{wp}\cdot \varepsilon_{wp}+m_{wi}\cdot \varepsilon_{wi}+\varepsilon_{q}+\varepsilon_{d}$$

where *m* is the mass flow expressed in kg/h, the subscripts *dp* indicate dry product, *ma*, (moist air) humid air, *wo* output humidity, *da* dry air or inlet air, *wp*, humid product and *wi*, input humidity.

Where *ε_d_* is the destroyed exergy and *ε_q_* is the exergy associated with heat losses in kJ/h. The destroyed exergy due to irreversibilities is calculated as

(3) $$\varepsilon_{d}=T_{0}\cdot S_{gen}$$

where *T*_0_ is the environment temperature, and *S_gen_* is the generated entropy in the process expressed in kJ/K.

The difference in the input and output exergy flows of the drying chamber is equal to the sum of the thermal exergy loss and the destroyed exergy due to irreversibility. The exergy flow of a stream in stationary flow is given by

(4) $$\dot{\varepsilon }=\dot{m}\left[Cp\left(T-T_{0}\right)-T_{0}\left\{Cpln\left(\frac{T}{T_{0}}\right)-Rln\left(\frac{P}{P_{0}}\right)\right\}\right]$$

where $\dot{\varepsilon }$ is the flow exergy (kJ/h), $\dot{m}$ is the mass flow (kg/h), *Cp* is the flow specific heat (kJ/kg K), R is the gases constant, *T*_0_ and *P*_0_ are the reference temperature and pressure considered as 298 K and 1 atm, respectively, *T* and *P* are the flow temperature and pressure.

The change in pressure between the inlet and outlet dryer flows is negligible. So, the Equation (4) can be written as

(5) $$\dot{\varepsilon }=\dot{m}Cp\left[\left(T-T_{0}\right)-T_{0}ln\left(\frac{T}{T_{0}}\right)\right]$$

Taking into account the moisture content of the drying air, its exergy is expressed as a function of the conditions at the inlet or outlet dryer flows such as

(6) $$\varepsilon_{da}=Cp_{da}\cdot \left(T-T_{0}\right)-T_{0}\left[Cp_{da}ln\left(\frac{T}{T_{0}}\right)\right]+T_{0}\left[Rln\left(\frac{1+1.6078\cdot \omega_{0}}{1+1.6078\cdot \omega }\right)+1.6078\omega Rln\left(\frac{\omega }{\omega_{0}}\right)\right]$$

where *Cp_da_* is the specific heat of the drying air (kJ/kg K), *ω* is the specific humidity (kg water/kg dry air), $\omega_{0}$ is the reference humidity (0.009 kg water/kg dry air). The specific heat of the drying air is calculated as [12]

(7) $$Cp_{da}=\frac{1.004+1.88\cdot \omega }{1000}$$

The physical exergy of the wet product can be expressed as

(8) $$\varepsilon_{p}=\left(H-H^{0}\right)-T_{0}\left(S-S^{0}\right)=\left[H_{p}\left(T,P\right)-H_{p}\left(T_{0},P_{0}\right)\right]-T_{0}\left[S_{p}\left(T,P\right)-S_{p}\left(T_{0},P_{0}\right)\right]$$

*T*_0_ and *P*_0_ are the temperature and pressure of environmental state taken as a reference, respectively. The temperature and pressure are equal to 298 K and 1 atm, respectively.

The onion specific heat as a function of moisture content is given by [13]

(9) Cp=1.84+2.34·w

where *w* is the water mass fraction in the onion.

The exergy losses in the drying chamber are calculated as [12]

(10) $$\varepsilon_{loss}=\varepsilon_{in}-\varepsilon_{out}=\sum^{\text{​}}\varepsilon_{L}=\sum^{\text{​}}\varepsilon_{i}-\sum^{\text{​}}\varepsilon_{o}$$

where $\varepsilon_{loss}$ is the total lost exergy (kJ/h), $\varepsilon_{i}$ is the exergy associated with the input flows at the drying chamber and $\varepsilon_{o}$ is the exergy associated with the output flows.

The exergy efficiency is defined as the ratio of the used exergy in the product drying and the exergy of the drying air supplied to the system.

(11) $$\eta_{\varepsilon x}=\frac{Exergy\;inflow-Exergy\;loss}{Exergy\;inflow}=\frac{\varepsilon_{i}-\varepsilon_{L}}{\varepsilon_{i}}=1-\frac{\varepsilon_{L}}{\varepsilon_{i}}$$

where $\eta_{\varepsilon x}$ is the is the exergy efficiency. The optimal values of this parameter for a system or process are obviously reached when the exergy losses are minimized. Considering that, a single exergetic indicator might not be sufficient to describe completely the thermodynamic performance of drying process, different parameters such as *IP* and *SI* were used.

The concept of *IP* is commonly used to analyze different processes or economic sectors. It is clear that maximum improvement in the $\eta_{\varepsilon x}$ for a process is reached when $\varepsilon_{L}$ is minimized. Therefore, different authors proposed the use of this concept to analyze a process [2,14,15]. IP is given by

(12) $$IP=\left(1-\eta_{\varepsilon x}\right)\varepsilon_{L}$$

On the other hand, it is important to take into account that to carry out the exergy analysis, usually, the temperature, pressure, and chemical composition of the reference environment must be specified. Its results are relative to this environment, generally the actual local environment. This bond between exergy and the environment has implications regarding the environmental impact. The exergy efficiency considers energy flows by counting for each in terms of accessibility. This analysis establishes that, to improve performance, both losses and internal irreversibilities must be attended to. The environmental impact factor of onion drying is a significant parameter to show whether or not it harms the environment due to the exergy destruction. So, it is possible to use exergy analysis to evaluate a system and its environmental impact. For that, the *SI* is defined as the relation between the input exergy and the exergy losses of the system. This index can obtain information about the process influence on the environment, and it is considered an important evaluation parameter [16]. The *SI* is calculated as

(13) $$SI=\frac{1}{1-\eta_{\varepsilon x}}$$

The *SI* is a function of the relationship between residual exergy and exergy efficiency. The environmental impact factor decreases if the *SI* increases [17].

## 3. Results and Discussion

**Figure 1:**
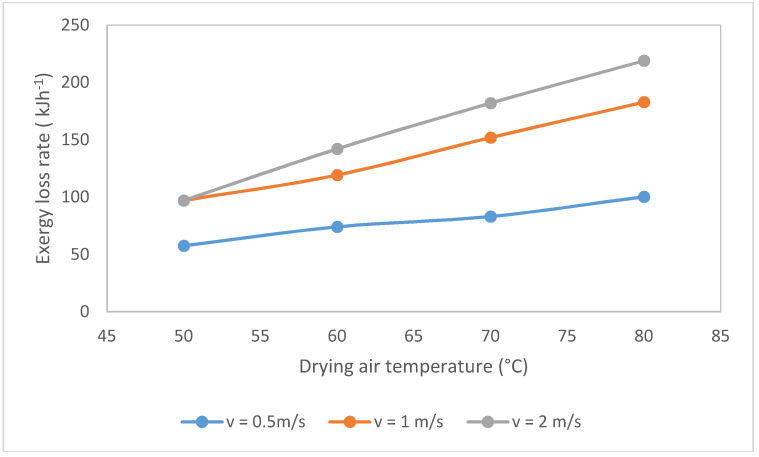
Exergy loss rate variation with the temperature and air velocity.

The calculation basis was equal to 1 kg of onion/h. Different temperatures and velocity of drying air were considered: 50, 60, 70, and 80 °C and 0.5, 1, and 2 ms^−1^, respectively. The exergy loss rate related to the onion drying increases with temperature and the velocity (Figure 1). The highest values of these rates are reached at velocity equal to 2 ms^−1^ and temperature equal to 80 °C. The losses rate value is 218.89 kJh^−1^, representing 28.6% of the whole inlet exergy at the drying chamber. It is important to take into account that one of the thermodynamic inefficiencies of these systems is the exergy loss from the drying chamber to the environment. With the temperature and velocity increase, the input exergy to the drying chamber grows, and then, a large amount of the input exergy comes out without evaporating moisture contained in the onion. Additionally, exergy loss rate is diminished at lower drying air temperatures and velocities because the overall heat transfer coefficient decreases [5]. Corzo et al. [12], Azadbakht et al. [9] and Nazghelichi [18] obtained analogous observations.

**Figure 2:**
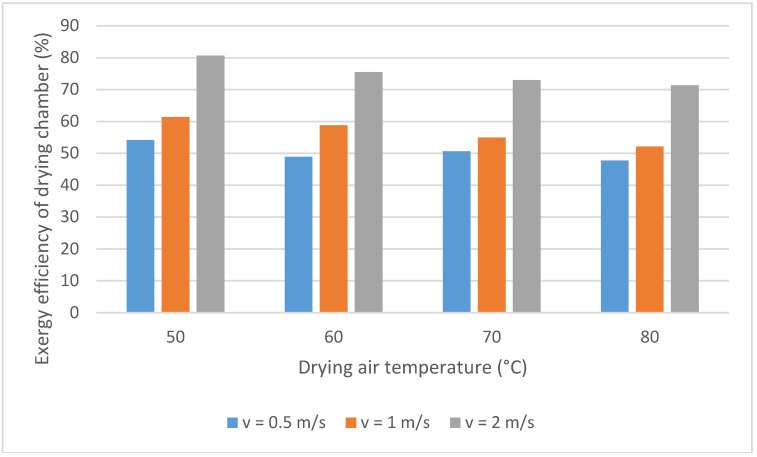
Exergy efficiency of the drying chamber. Variation with the temperature and air velocity.

Figure 2 shows the exergy efficiency variation of the drying chamber with the analyzed variables. As it can be observed, the exergy efficiency increases with the air velocity augmentation. Aghbashlo et al. [19] obtained similar results. The energy use was increased due to the most amount of supplied energy was utilized for the moisture evaporation. Heat loss augmented when the velocity increased, probably due to growth in the overall heat transfer coefficient. However, these losses are very small compared with the effect of the inlet enthalpy on heat and mass transfer, causing a high energy utilization. In addition, at higher air velocities, there is an increase in the output exergy comparing with the input air exergy. So, the exergy efficiency is equal to 50.6%, 54.9%, and 73.0% for a temperature of 60 °C and air velocity equal to 0.5, 1, and 2 ms^−1^, respectively; demonstrating that the convective drying is a process moderately poor, considering the exergy efficiency.

The process exergy efficiency decreased by the augmentation in the inlet drying air temperature. Similar results were obtained by [7,14,15,20]. However, other authors [19,21] observed that the exergy efficiency increased with the temperature augmentation; this can be due to moisture saturated surface for which more heat is utilized to evaporate the free moisture. Yet, when this moisture is less, the moisture begins diffusing from the internal structure to the surface [7].

According to the proposed model, for a drying air temperature and velocity equal to 50 °C and 2 ms^−1^, respectively, the calculated exergy efficiency was 80.7%. Aghbashlo et al. [19] obtained values close to 87% and the values obtained for Khanali et al., 2013 varied between 65% and 74%, approximately. For a drying temperature equal to 70 °C, the exergy efficiency was about 73%, and these researchers found values about 67%.

**Figure 3:**
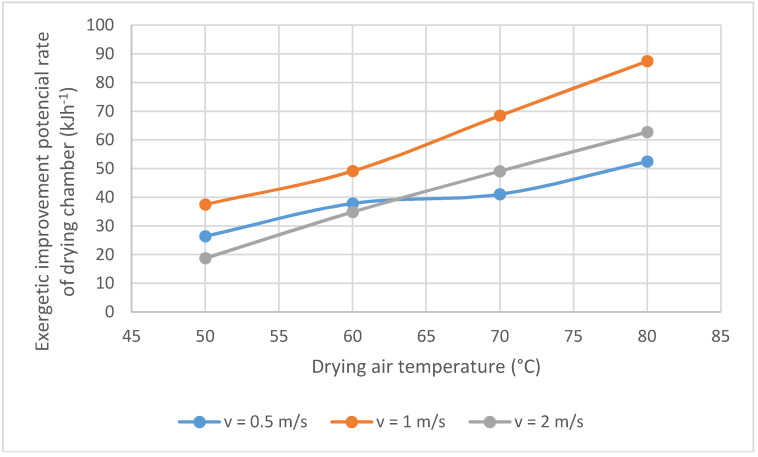
Exergetic improvement potential rate. Variation with the temperature and air velocity.

Figure 3 shows the variation of *IP* rate in the onion drying chamber with temperature and air velocity. Their values vary from 18.7 to 87.4 kJh^−1^. The *IP* rate, under studied operative conditions, vary from 21–29.9% of the total input exergy. These values show that the exergy efficiency of onion drying process can be ameliorated. Aghbashlo et al. [14] founded *IP* rate values between 37.21% and 73.2% to fish oil microencapsulation process by spray drying. Beigi et al. [22] reported values between 27.3% and 59.21% of this parameter for the deep-bed drying of rough rice. Instead, its value increases when the temperature. However, the influence of the air velocity is not clear. Similar results were found by Kuzgunkaya and Hepbasli [23] by Icier, et al. [24]. Considering that the exergy is conserved only for reversible processes, the growth in exergy destruction moves away the process of reversibility, causing *IP* rate increase. The onion drying process, consequently, presents a potential for exergy efficiency growth. It is important to note that different efforts should be oriented to improve the exergy efficiency of the studied process. In this case, considering the studied ranges of the operation variables, onion drying could be carried out at a temperature equal to 50 °C and an air velocity equal to 2 m/s.

**Figure 4:**
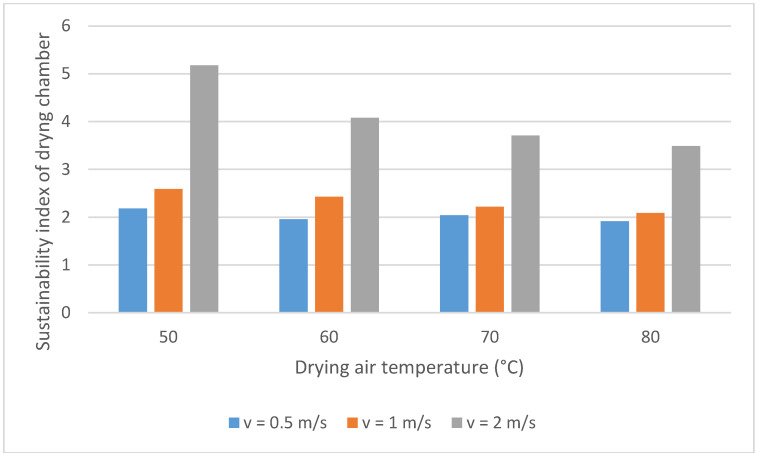
Sustainability index of drying chamber. Variation with the temperature and air velocity.

The effect of temperature and air velocity on the *SI* of the onion drying chamber can be seen in Figure 4. At higher exergy efficiency values, the sustainability index increases and the environmental impact will be lower. The *SI* varies from 1.9 to 5.1 under studied operating conditions. Beigi et al. [22] calculated *SI* and founded values between 1.48 and 3.11. Its value increase with the air velocity augmentation, however, it diminishes with the temperature increase. The influences of these parameters on the *SI* and exergy efficiency drying chamber are similar. It is important to note that the highest *SI* values show a low environmental impact. Therefore, in order to reduce this impact, the exergy efficiency should be improved.

## 4. Conclusions

The influence of drying air parameters in an onion drying system was studied in terms of exergy parameters. The temperature and air velocity were considered in order to carry out this analysis. A thermodynamic model was proposed based on the matter, energy, and exergy balances taking into account the onion drying chamber. The chamber behavior was analyzed based on exergy efficiency, exergy loss rate, *IP* rate, and *SI*.

The exergy loss rates increase with the temperature and air velocity augmentation. The minimum value is 57.5 kJ/h at 50 °C and 0.5 m/s.

The exergy efficiency increases with the air velocity augmentation and decreases with the temperature. The maximum value to this parameter was obtained at 50 °C and 2 m/s being equal to 80.6%.

The *IP* rates, under studied operative conditions, vary from 21–29.9% of the total input exergy. This parameter augments with the temperature. However, the influence of air velocity is not clear. This process shows a lower *IP* than fish oil microencapsulation process by spray drying and deep-bed drying of rough rice.

The *SI* of the drying chamber varied from 1.9 to 5.1. This drying process shows better *SI* than deep-bed drying of rough rice. To reduce the process environmental impact, the parameters must be modified in order to ameliorate the exergy efficiency of the drying process. 

*PI* and *SI* values show that better drying conditions are coincident with those presented by exergy efficiency.

## Figures and Tables

**Table 1:** The variation of air flow rate at different conditions

Air Velocity (m/s)	Inlet Air Temperature (°C)	Air density (kg/m^3^)	Mass Air Flow Rate (kg/h)	Volumetric Air Flow Rate (m^3^/h)
0.5	50	1094	122.38	0.11
	60	1061	73.99	0.07
	70	1030	52.99	0.05
	80	1001	41.25	0.04
1	50	1094	245.06	0.22
	60	1061	147.48	0.14
	70	1030	106.09	0.10
	80	1001	82.08	0.08
2	50	1094	490.11	0.45
	60	1061	294.96	0.28
	70	1030	212.18	0.21
	80	1001	164.16	0.16
